# A Nanoparticulate Ferritin-Core Mimetic Is Well Taken Up by HuTu 80 Duodenal Cells and Its Absorption in Mice Is Regulated by Body Iron^1^,^2^

**DOI:** 10.3945/jn.114.201715

**Published:** 2014-10-23

**Authors:** Gladys O Latunde-Dada, Dora IA Pereira, Bethan Tempest, Hibah Ilyas, Angela C Flynn, Mohamad F Aslam, Robert J Simpson, Jonathan J Powell

**Affiliations:** 3 Diabetes and Nutritional Sciences Division, Faculty of Life Sciences and Medicine, King's College London, London, United Kingdom; and; 4 Medical Research Council Human Nutrition Research, Elsie Widdowson Laboratory, Cambridge, United Kingdom

**Keywords:** cellular uptake, iron absorption, iron deficiency anemia, iron supplementation, tartrate-modified Fe(III) poly oxo-hydroxide

## Abstract

**Background:** Iron (Fe) deficiency anemia remains the largest nutritional deficiency disorder worldwide. How the gut acquires iron from nano Fe(III), especially at the apical surface, is incompletely understood.

**Objective:** We developed a novel Fe supplement consisting of nanoparticulate tartrate-modified Fe(III) poly oxo-hydroxide [here termed nano Fe(III)], which mimics the Fe oxide core of ferritin and effectively treats iron deficiency anemia in rats.

**Methods**: We determined transfer to the systemic circulation of nano Fe(III) in iron-deficient and iron-sufficient outbread Swiss mouse strain (CD1) mice with use of ^59^Fe-labeled material. Iron deficiency was induced before starting the Fe-supplementation period through reduction of Fe concentrations in the rodent diet. A control group of iron-sufficient mice were fed a diet with adequate Fe concentrations throughout the study. Furthermore, we conducted a hemoglobin repletion study in which iron-deficient CD1 mice were fed for 7 d a diet supplemented with ferrous sulfate (FeSO_4_) or nano Fe(III). Finally, we further probed the mechanism of cellular acquisition of nano Fe(III) by assessing ferritin formation, as a measure of Fe uptake and utilization, in HuTu 80 duodenal cancer cells with targeted inhibition of divalent metal transporter 1 (DMT1) and duodenal cytochrome b (DCYTB) before exposure to the supplemented iron sources. Differences in gene expression were assessed by quantitative polymerase chain reaction.

**Results:** Absorption (means ± SEMs) of nano Fe(III) was significantly increased in iron-deficient mice (58 ± 19%) compared to iron-sufficient mice (18 ± 17%) (*P* = 0.0001). Supplementation of the diet with nano Fe(III) or FeSO_4_ significantly increased hemoglobin concentrations in iron-deficient mice (170 ± 20 g/L, *P* = 0.01 and 180 ± 20 g/L, *P* = 0.002, respectively). Hepatic hepcidin mRNA expression reflected the nonheme-iron concentrations of the liver and was also comparable for both nano Fe(III)– and FeSO_4_-supplemented groups, as were iron concentrations in the spleen and duodenum. Silencing of the solute carrier family 11 (proton-coupled divalent metal ion transporter), member 2 (*Slc11a2*) gene (DMT1) significantly inhibited ferritin formation from FeSO_4_ (*P =* 0.005) but had no effect on uptake and utilization of nano Fe(III). Inhibiting DCYTB with an antibody also had no effect on uptake and utilization of nano Fe(III) but significantly inhibited ferritin formation from ferric nitrilotriacetate chelate (Fe-NTA) (*P* = 0.04). Similarly, cellular ferritin formation from nano Fe(III) was unaffected by the Fe(II) chelator ferrozine, which significantly inhibited uptake and utilization from FeSO_4_ (*P* = 0.009) and Fe-NTA (*P* = 0.005).

**Conclusions:** Our data strongly support direct nano Fe(III) uptake by enterocytes as an efficient mechanism of dietary iron acquisition, which may complement the known Fe(II)/DMT1 uptake pathway.

## Introduction

Iron deficiency and iron deficiency anemia (IDA)^5^ are nutritional disorders afflicting a substantial proportion of the world’s population (1). These problems are most prevalent among vulnerable infants, adolescent girls, pregnant women, and the elderly in both developed and developing countries. Aside from the high iron demand posed by increased physiologic requirements of growth and reproduction, IDA is further accentuated by inadequate dietary iron intake and poor iron bioavailability from food sources (2). Debilitating effects of IDA on cognition, work performance, and pregnancy outcomes have been reported (3, 4). The types of iron compounds that are used for food fortification and/or supplementation include simple Fe(II) salts (e.g., ferrous sulfate, ferrous fumarate, ferrous gluconate); Fe(III) minerals/salts (e.g., ferric pyrophosphate, ferric ammonium citrate); Fe(III) chelates (e.g., sodium ferric EDTA, ferric citrate); and elemental Fe powders (e.g., electrolytic iron, carbonyl iron). Simple Fe(II) salts remain the oral iron compounds of choice (5, 6) despite their well-recognized toxicity, which leads to gastrointestinal side effects, compromised compliance, and additional healthcare costs (7–10). Moreover, current iron supplements might induce DNA damage in the gastrointestinal tract by generating reactive oxygen species through the Fenton reaction (11–13). Another possible mechanism contributing to gastrointestinal side effects is through iron-induced changes to the composition of the colonic microflora (13–15). Finally, there are substantial concerns that soluble Fe(III) chelates may directly increase tumor burden in colorectal carcinogenesis, despite not being active in redox cycling (16, 17).

We developed a nano particulate ligand-modified Fe(III) poly oxo-hydroxide [i.e., tartrate-modified ferrihydrite, here termed nano Fe(III)] that mimics the ferrihydrite core of ferritin and that may find use in the prophylactic and therapeutic management of IDA (18). Moreover, nano Fe(III) resembles the iron species naturally formed in the intestinal lumen during digestion of dietary nonheme iron (19). We aimed to investigate the following: *1*) the impact of body iron status on iron absorption from nano Fe(III) in outbread Swiss mouse strain (CD1) mice, which are widely used in studies of iron metabolism (20–23); *2*) the efficacy of nano Fe(III) in treating iron deficiency anemia in comparison with gold standard oral ferrous sulfate in the same mouse model; and *3*) the use of small interfering RNA (siRNA), antibodies, and iron chelation to provide novel insights into the acquisition of nano iron (III) by a duodenal epithelial cell line (HuTu 80).

## Methods

Fe(II) sulfate heptahydrate (FeSO_4_) and ferric ammonium citrate (FAC) were purchased from Sigma-Aldrich. Fe(III) nitrilotriacetate chelate [Fe(III)-NTA] was produced by mixing a solution of Fe(III) chloride (10 mmol/L) with a NTA solution to achieve a molar ratio of Fe:NTA of 1:2. FeSO_4_-ascorbate was prepared by mixing FeSO_4_ and ascorbic acid in a molar ratio of 1:1 and [Fe] = 20 mmol/L. Ligand-modified Fe(III) poly oxo-hydroxide, here referred to as nano Fe(III), was produced following the protocol described by Powell et al. (18). Briefly, an acidic concentrated stock solution of Fe(III) chloride was added to a solution containing adipic and tartaric acids to obtain a final iron concentration of 40 mmol/L and a molar ratio of Fe:adipic:tartaric = 2:1:1. The initial pH of the mixture was <2.0, and the iron was fully solubilized as determined by ultrafiltration (M_r_ 3000 molecular weight cut-off; 10,000 ×* g*, 10 min). The pH was then slowly increased by drop-wise addition of a concentrated solution of NaOH with constant agitation until approximately pH 7.4. The entire mixture was then oven-dried at 45°C for a minimum of 24 h.

### Animal studies

CD1 strain male mice (Charles Rivers) were used for the studies and were 3 wk of age when weaned onto the test diets. Mice were housed in a light- and temperature-controlled room with ad libitum access to standard pellet diet and water. Animal care and all procedures were conducted in accordance with methods approved by the United Kingdom Animals (Scientific Procedures) Act 1986.

#### Hemoglobin repletion study.

Twelve male CD1 mice (3 wk of age) were made Fe deficient through use of a low-iron diet of ∼3-mg Fe/Kg diet based on the modified AIN-76A purified rodent diet (24) (TD.80396; Harlan Teklad) for 3 wk (i.e., until they were 6 wk of age). Four mice were also placed on a normal iron-sufficient diet (48-mg Fe/Kg diet) (TD.80394; Harlan Teklad) to serve as control. The diets were of identical composition except that the iron-sufficient diet contained iron added as ferric citrate. After this, blood was withdrawn from the tails to determine the initial Hb concentrations of the mice. The Fe-deficient mice were then divided into 3 treatment groups based on similar Hb concentrations. These 12 mice were maintained on the low-Fe diet in groups of 4, of which 1 group did not receive any iron supplementation (low-iron diet); the 2 other groups were gavaged daily with 150-μg Fe as nano Fe(III) compound or FeSO_4_ for 7 d (until mice were 7 wk of age). After the 7 d, mice were weighed, anesthetized, and blood samples were taken for Hb and serum iron determinations. The mice were then killed by isoflurane anesthesia followed by neck dislocation, and the spleen, duodenum, kidney, and liver samples were excised, snap frozen in liquid nitrogen, and stored at −80°C until further analysis.

#### ^59^Fe absorption study.

Iron absorption was measured in control iron-sufficient mice (fed an iron-sufficient diet for 3 wk, i.e., mice were 6 wk of age) and in iron-deficient mice (low-iron diet for 3 wk) that were feed-deprived for ∼16 h before given a test dose containing 20 mmol/L Fe as nano ^59^Fe(III) or ^59^FeSO_4_-ascorbate. The test doses (100 μL) were administered by oral gavage into the stomach (112 μg Fe). Mice were left for 4 h (25) with free access to drinking water until they were killed and tissue was collected as described by Simpson and Peters (26). Total unabsorbed radioiron was defined as the sum of the amounts of radio-iron in duodenal washes, and jejunum, ileum, and colonic tissue. All values are expressed as a percentage of administered dose.

### Cell studies

Duodenal HuTu 80 cells (a human epithelial adenocarcinoma adherent cell line) were obtained from the American Type Culture Collection. HuTu 80 cells do not differentiate with enterocyte-like properties such as Caco-2 cells or form a polarized monolayer, but their siRNA transfection efficiency is less variable. Cells were cultured in DMEM (Life Technologies) supplemented with 10% fetal calf serum (Sigma-Aldrich) and with 100-kU/L penicillin and 100-mg/L streptomycin. Cells were maintained at 37°C in an atmosphere of 5% CO_2_ and 95% air at a relative humidity of ∼95%. Cells were passaged at 70% confluence with use of GibcoVersene Solution (Life Technologies).

#### siRNA transfection.

Small interfering RNA oligonucleotides were purchased from Invitrogen Life Technologies. Cells were transfected with 10 nM of either the Silencer Select Negative Control No. 1 (4390843, scramble) or Silencer Select siRNA targeting *Slc11a2* (S9708). HuTu 80 cells at 50% confluence (24-h postseeding) were transfected with siRNAs with use of RiboCellin transfection reagent (BioCell Challenge), following protocols provided by the manufacturer. Transfected cells were assayed after 72 h for divalent metal transporter 1(DMT1) mRNA expression by qRT-PCR and for iron uptake as detailed below. Antibody inhibition, targeting duodenal cytochrome b (DCYTB) (cytochrome b reductase 1 polyclonal; Novus Biologicals) in HuTu 80 cells, was carried out with use of ImmunoCellin live cells antibody transfection reagent (BioCell Challenge) according to manufacturer protocols.

#### Iron uptake.

To avoid aggregation/agglomeration of the nano Fe(III), the medium used for cellular uptake consisted of a balanced salt solution containing 130-mmol/L NaCl, 10-mmol/L KCl, 1-mmol/L MgSO_4_, 5-mmol/L glucose, and 1-mmol/L CaCl_2_ in 10-mmol/L PIPES buffer (pH 6.5) (27). Unless otherwise stated, the iron concentration was 10 μmol/L. Confluent HuTu 80 cells were exposed to serum-free DMEM for 4 h before iron uptake studies as described by Pereira et al. (19). Cells were then incubated for 1 h with 50- or 100-μmol/L [Fe] as nano Fe(III), FeSO_4_, Fe-NTA, or FAC in balanced salt solution, plus 23 h in fresh nonsupplemented DMEM. To investigate the effect of ferrozine (1 mmol/L) or zinc (50 μmol/L as ZnSO_4_) on cellular ferritin formation from nano Fe(III), FeSO_4_, Fe-NTA, or FAC, cells were co-incubated with 10-μmol/L Fe and the chemical inhibitor for 1 h followed by 23 h in fresh nonsupplemented DMEM. The cell lysates were collected and used for ferritin and protein analysis as previously described (19). Experiments were carried out in triplicate and data expressed as nanogram ferritin per milligram cell protein.

### Analysis

#### Hematology.

Hemoglobin concentrations were calculated from the change in optical density at 540 nm, after the addition of 5 μL of whole-blood to Drabkin’s reagent (Sigma-Aldrich) and centrifugation (Heraeus Biofuge Pico) at 16,060 ×* g* for 5 min.

#### Tissue nonheme iron.

Tissue samples were weighed and homogenized (1:5 wt:vol) in 0.15-mol/L NaCl in 10-mmol/L NaOH-Hepes buffer (pH 7.0) with use of a 1-mL glass dounce homogenizer (Wheaton Scientific). An aliquot of the homogenate was then analyzed for nonheme-iron content with use of the ferrozine-based colorimetric assay developed by Simpson and Peters (27). The iron values were expressed as either content (μmol Fe/organ) or concentration (nmol Fe/mg wet weight).

#### Real-Time PCR.

Total RNA was extracted from tissue samples through use of TRIZOL reagent (Invitrogen) according to the manufacturer’s instructions. To determine hepcidin antimicrobial peptide 1 (*Hamp1*)- expression, quantitative RT-PCR was carried out with use of an ABI Prism 7000 detection system (Applied Biosystems) in a 2-step protocol with Roche Universal primers and probes. Quantitative measurement of each gene was normalized to the threshold cycle value for mouse ribosomal protein L19 (*Rpl19*).

Sequences of primers used are as follows (5′-3′):

- *Rpl19*, forward CTCGTTGCCGGAAAAACA
- *Rpl19*, reverse TCATCCAGGTCACCTTCTCA
- Mouse *Hamp1*, forward AGAAAGCAGGGCAGACATTG
- Mouse *Hamp1*, reverse CACTGGGAATTGTTACAGCATT

For the siRNA cell transfection experiments, *Slc11a2* (DMT1) expression was determined with use of TaqMan Gene Expression Assay Master Mix (Applied Biosystems), according to the manufacturer’s protocol, on an Applied Biosystems 7500 fast real-time PCR system using probe ID: Hs00167206_m1 (5′-3′ TGTGTTCTACTTGGGTTGGCAATGT). Expression was relative to the *18S* ribosomal RNA (*18S*) housekeeping gene, and the percent knockdown was calculated with use of the ΔΔCq method.

#### Ferritin ELISA assay in cell lysates.

The Spectro Ferritin MT ELISA kit (ATI Atlas) was used to determine cellular ferritin content as described in the manufacturer’s protocol. Cellular protein concentration was determined according to Bio-Rad assay protocol (Bio-Rad Laboratories). Experiments were carried out in triplicates, and data are expressed as nanogram ferritin per milligram protein.

### Statistical analysis

Unless otherwise indicated, values are means ± SEMs, with the number of independent experiments given in the figure legends. One-factor ANOVA with the Tukey’s test for multiple comparisons was used to compare means for hemoglobin concentrations, *Hamp1*-expression, and tissue (i.e., hepatic, splenic, duodenal) iron concentrations between the different diet groups. In the mouse study, 2-factor ANOVA was used to test the main effects and the interaction between radioiron in each body compartment and iron status (i.e., Fe-deficient and Fe-sufficient). Tukey’s post hoc test was used to determine the significant differences between radioiron absorption for nano Fe(III) and FeSO_4_ in Fe-sufficient vs. Fe-deficient mice for each body compartment. In the cellular assays, 2-factor ANOVA was used to test the main effects and the interaction between the iron compound and the experimental treatments. Tukey’s post hoc test was used to determine significant differences in ferritin formation between the different iron compounds in the different experimental conditions. Specific comparisons were the following: ferritin formation for each compound between the 2 Fe concentrations, ferritin formation for each compound with and without the inhibitory factor (namely ferrozine, zinc, DCYTB antibody, and *Slc11a2* siRNA), and ferritin formation under each experimental condition (i.e., Fe concentration and absence or presence of the inhibitory factor) between the different iron compounds.

Differences were considered statistically significant at *P* < 0.05. All statistical analyses were performed with use of GraphPad Prism 6.02 for Windows (GraphPad Software).

## Results

### 

#### In vivo absorption of nano Fe(III) after gavage.

**FIGURE 1:**
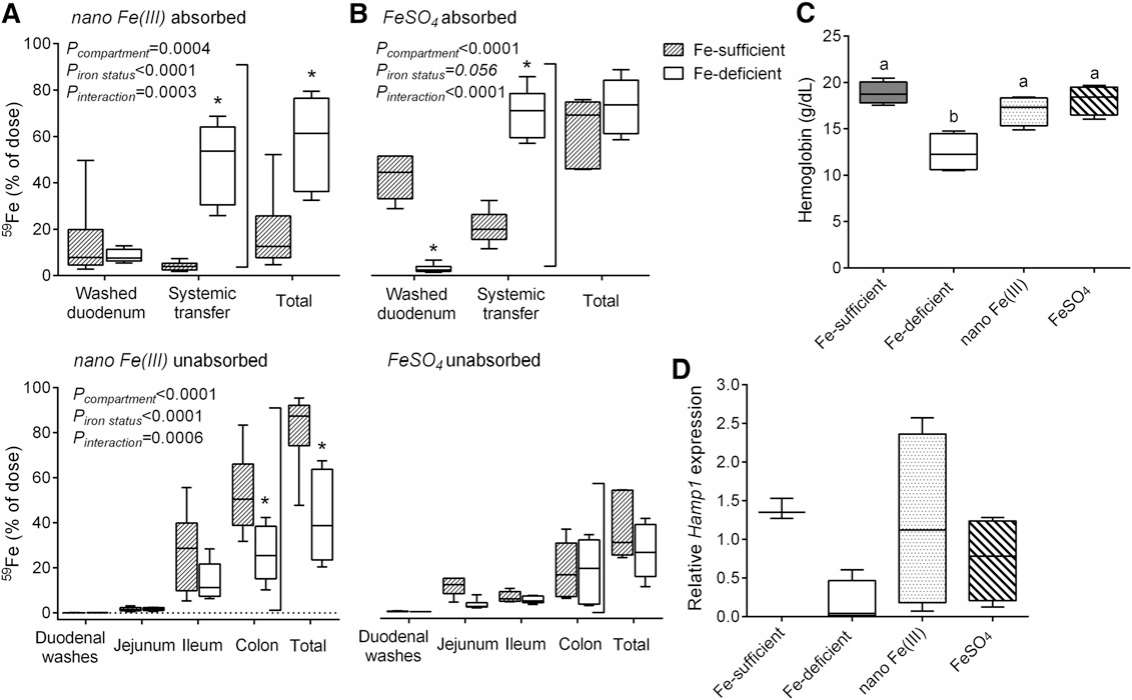
In vivo bioavailability of nano Fe(III) and FeSO_4_ in male CD1 mice. Effect of iron status on the absorption of nano Fe(III) (A) or FeSO_4_ (B) in mice after oral gavage with ^59^Fe-labeled material. Box and whisker plots show median, minimum, and maximum (*n* = 6 per group). All values are expressed as percentage of dose (i.e., the radio iron that has left the stomach). *Different from the Fe-sufficient control within each body compartment, *P* < 0.05 (2-factor ANOVA). Hemoglobin concentrations (C) and *Hamp1* mRNA expression (D) of mice after 7-d feeding with test diets supplemented with nano Fe(III) or FeSO_4_. Concentrations in control mice maintained in the Fe-sufficient or the Fe-deficient diets throughout the study are also shown. Box and whisker plots show median, minimum, and maximum (*n* = 3 in the Fe-deficient group; *n* = 4 in each of the other groups). Labeled means without a common letter differ, *P* < 0.05 (1-factor ANOVA). CD1, outbread Swiss mouse strain; FeSO_4_, ferrous sulfate;* Hamp1*, hepcidin antimicrobial peptide 1.

First we were interested in assessing impact of systemic iron status on iron absorption of nano Fe(III). Hence, we compared iron absorption 4 h after gavage with a single dose of ^59^Fe- labeled FeSO_4_ or nano Fe(III) in iron-deficient and iron-sufficient mice (**Figure 1A, B**). As expected, iron absorption beyond gut uptake (i.e., systemic transfer) was significantly lower (*P* < 0.0001) in iron-sufficient mice than iron-deficient mice for FeSO_4_ (Figure 1B). There was also significantly higher systemic transfer of iron from nano Fe(III) in iron-deficient mice than iron-sufficient mice (*P* < 0.0001), but the iron retained in the duodenal tissue was not significantly different (Figure 1A). Iron retained in the jejunum, ileum, or colon would normally be considered as unabsorbed iron (i.e., in transit). Indeed, almost all of the unabsorbed iron from nano Fe(III) in iron-sufficient mice could be accounted for in the ileal and colonic samples (Figure 1A). Over the 4-h period, 49 ± 17% of the gavaged iron was transferred systemically for nano Fe(III) vs. 70 ± 11% for ferrous sulfate (*P* = 0.03), although absolute comparisons in absorption are not easy because of likely differences in kinetics of uptake as observed in humans (28).

#### In vivo bioavailability of nano Fe(III) in a feeding study.

Next we considered efficacy at repleting hemoglobin concentrations of nano Fe(III) vs. FeSO_4_ after diet-induced iron deficiency. Mice fed a diet low in Fe (Fe-deficient group) for 4 wk showed significantly lower blood hemoglobin concentrations than control mice kept on the iron-sufficient diet (Fe-sufficient group) throughout the study (*P* = 0.0007, Figure 1C). Supplementation of the Fe-deficient diet with either nano Fe(III) or FeSO_4_ for 7 d significantly increased hemoglobin concentrations in comparison with mice fed the Fe-deficient diet throughout the study (*P* = 0.01 and 0.002, respectively, Figure 1C). *Hamp1* mRNA expression tended to be lower (*P* = 0.1) in Fe-deficient mice than Fe-sufficient mice (Figure 1D). Mice fed the diets supplemented with either nano Fe(III) or FeSO_4_ had hepatic *Hamp1* mRNA concentrations similar to control Fe-sufficient mice (*P* ≥ 0.6, Figure 1D). There were no significant differences among the diet groups in food intake or body weight throughout the study (data not shown).

#### Tissue iron distribution.

**FIGURE 2:**
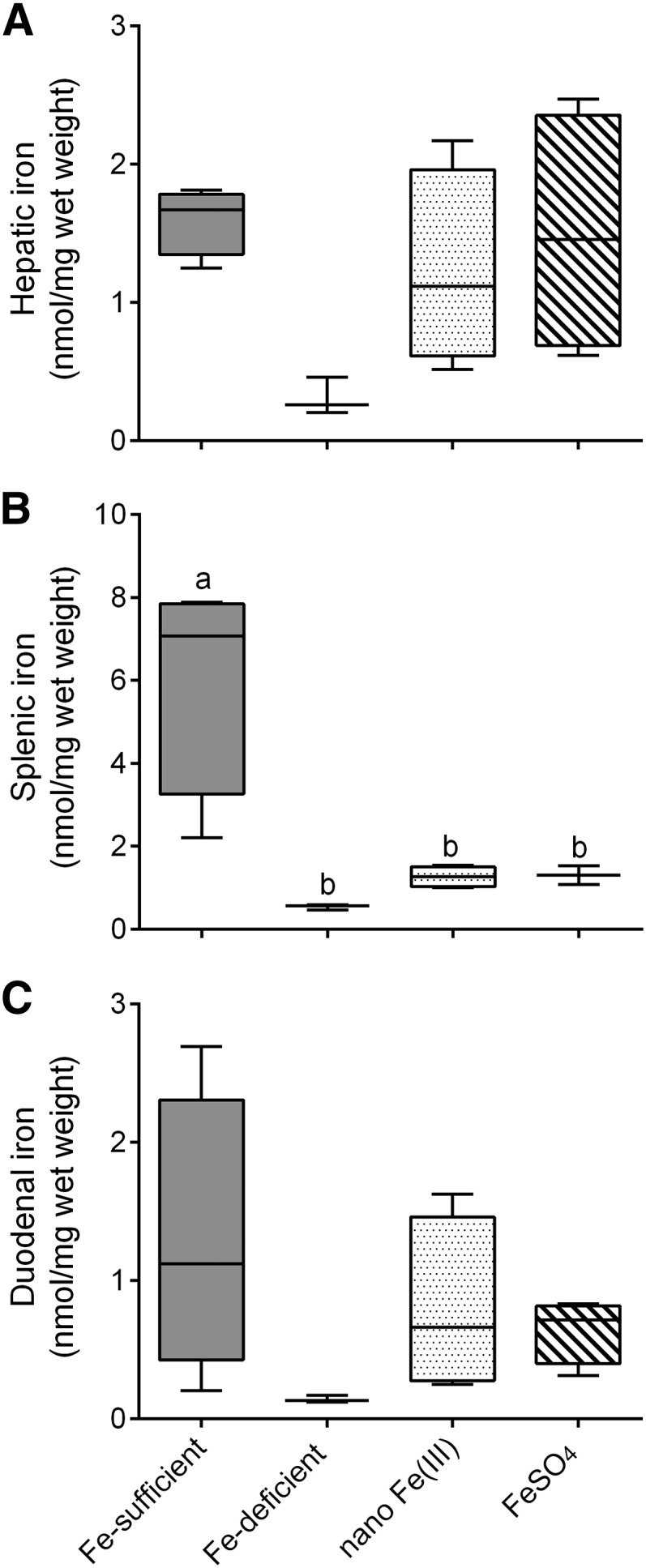
Tissue Fe distribution in male CD1 mice after supplementation with nano Fe(III) and FeSO_4_. Nonheme-iron concentrations in the liver (A), spleen (B), and duodenum (C) of mice after 7-d feeding with test diets supplemented with nano Fe(III) or FeSO_4_. Concentrations in control mice maintained in the Fe-sufficient or the Fe-deficient diets throughout the study are also shown. Box and whisker plots show median, minimum, and maximum (*n* = 3 in the Fe-deficient group; *n* = 4 in each of the other groups). Labeled means without a common letter differ, *P* < 0.05 (1-factor ANOVA). FeSO_4_, ferrous sulfate.

Feeding the Fe-deficient diet for 4 wk reduced the nonheme-Fe concentration in the spleen (*P* = 0.003) and tended to reduce it in the liver (*P* = 0.07) of Fe-deficient mice compared with Fe-sufficient mice (**Figure 2**). The concentration in the duodenum did not differ among groups. Final hepatic and duodenal nonheme-Fe concentrations did not differ after 7 d of iron supplementation in the test diets with either nano Fe(III) or FeSO_4_ (Figure 2), and this was reflected by similar concentrations of *Hamp1* mRNA in the 2 Fe-supplemented groups (Figure 1D). Nonheme-Fe concentrations in the spleen were still significantly lower than those in Fe-sufficient control mice for both nano Fe(III)- (*P* = 0.004) and FeSO_4_-supplemented (*P* = 0.008) groups and for the group maintained on the Fe-deficient diet throughout the study (*P* = 0.003) (Figure 2B).

#### Iron uptake mechanism in HuTu 80 cells.

**FIGURE 3:**
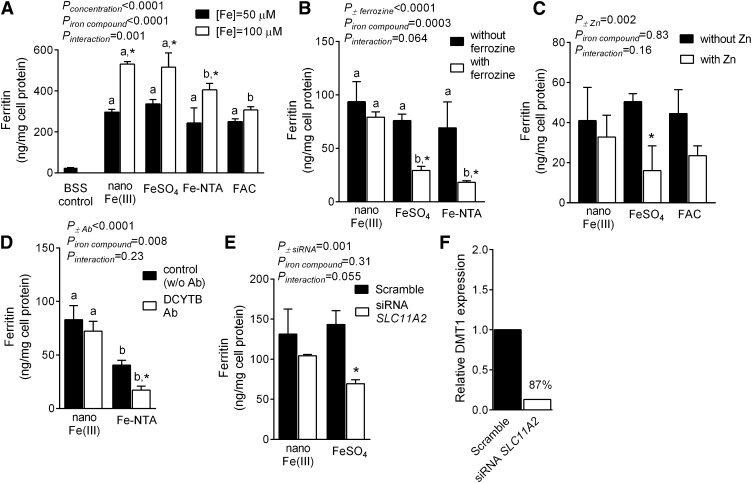
Uptake and utilization of nano Fe(III) in HuTu 80 cells. (A) Cellular ferritin concentrations in HuTu 80 cells after exposure to 50- or 100-μmol/L [Fe] as nano Fe(III), FeSO_4_, Fe-NTA, or FAC. Baseline ferritin concentrations in control cells incubated only with the BSS vehicle (gray bar) were ∼26 ng/mg cell protein and are shown for reference. Effect of ferrozine (B) or zinc (C) on cellular ferritin formation from nano Fe(III), FeSO_4_, Fe-NTA, or FAC. Effect of Ab inhibition targeting DCYTB (D) or siRNA targeting *Slc11a2* (E) on nano Fe(III) uptake and utilization in HuTu 80 cells. (A–E) Values are means ± SDs of 3 independent experiments (each experiment with 3 replicate wells). Means for each experimental condition (i.e., [Fe] concentration in A or inhibitor/knockdown treatment in B–E) without a common letter differ, *P* < 0.05. *Different from the corresponding control (closed bars) for each iron compound (2-factor ANOVA). (F) Relative DMT1 gene expression (*Slc11a2*) from qPCR data with *18S* ribosomal RNA as the endogenous housekeeping gene. Values are the mean of 2 replicates in the same experiment and percentage knockdown is shown relative to the nontargeting scramble. Ab, antibody; BSS, balanced salt solution; DCYTB, duodenal cytochrome b; DMT1, divalent metal transporter 1; FAC, ferric ammonium citrate; Fe-NTA, ferric nitrilotriacetate chelate; FeSO_4_, ferrous sulfate; siRNA, small interfering RNA; *Slc11a2*, solute carrier family 11 (proton-coupled divalent metal ion transporter), member 2.

Ferritin formation by HuTu 80 cells was used to assess cellular uptake and utilization from nano Fe(III) in comparison to FeSO_4_, Fe-NTA, or FAC. Ferritin formation after 1 h of exposure to 50- or 100-μmol/L Fe increased with dose for all compounds investigated, and this was significant for nano Fe(III) (*P* < 0.0001), FeSO_4_ (*P* = 0.0009), and Fe-NTA (*P* = 0.002) (**Figure 3A**). There was no difference in Fe utilization after exposure of cells to nano Fe(III) or FeSO_4_, but Fe utilization after Fe-NTA or FAC was significantly lower (*P* ≤ 0.02) (Figure 3A). In contrast to soluble Fe(III) (Fe-NTA), cellular uptake and utilization of nano Fe(III) by HuTu cells did not require prior reduction of Fe(III) to Fe(II) as shown by Fe(II)-chelation with ferrozine and by DCYTB antibody inhibition studies (Figure 3B, D). We also used an excess of Zn (5:1), and siRNA targeting *Slc11a2*, to probe the involvement of DMT1 on the apical uptake of iron from FeSO_4_ and nano Fe(III) in HuTu cells. In contrast to findings with FeSO_4_ (*P* = 0.02 with Zn and *P* = 0.005 with siRNA), there were no significant differences in ferritin formation after nano Fe(III) with or without Zn or siRNA transfection to inhibit DMT1, i.e., apical uptake of iron from nano Fe(III) in these cells was independent of DMT1 (Figure 3C, E). Calculations from qPCR data with use of the ΔΔCq method showed 87% knockdown of DMT1 when cells were treated with the siRNA targeting *Slc11a2* [normalized to *18S* housekeeping gene (Figure 3F)].

## Discussion

Food iron fortification and the use of iron supplements are approaches that have been adopted by several countries to try to combat iron deficiency and IDA and improve iron nutrition (23, 24). Inexpensive simple ferrous salts continue to be the standard for oral iron supplementation and fortification despite the many concerns associated with gastrointestinal tolerability and toxicity (5, 6, 11, 29). This is because alternative forms of oral iron, that are currently in use or are being developed, may have benefit in terms of improved tolerance but not demonstrably so in safety, and are mostly expensive (30, 31). Thus, even if they are well absorbed, they are often labile in the gut lumen or at the mucosal surface and available for bacterial uptake (13, 15, 32) or for the generation of toxic reactive oxygen species with potential detrimental effects (12, 13). Chelated iron is a particular concern with its effects on amplification of colon cancer risk (16, 17).

We are attempting to address the unmet clinical need for an economical, effective, safe, and tolerated form of oral iron that will correct IDA without side effects. To meet this challenge we have developed a mimetic of the form of iron that occurs naturally in the gut lumen upon digestion of nonheme-food iron. This synthetic mimetic is a nano particulate tartrate-modified Fe(III) poly oxo-hydroxide [termed here nano Fe(III)]; it is bioavailable in humans (28) and efficacious at treating IDA in rats (18). We have also shown in a mouse model that systemic transfer of iron, derived from nano Fe(III), is ferroportin-mediated (33). With the studies reported herein we add to this knowledge and now show that, in mice, the absorption of nano Fe(III) is regulated by systemic iron status (Figure 1) similarly to soluble iron species (34–36). Importantly, to our knowledge, this is the first report to show that nano Fe(III) is under normal iron homeostasis in wild-type mice of varying Fe status. Moreover, we provide some evidence of a further homeostatic step for iron absorption from nano Fe(III) in vivo because enterocyte retention of iron, in iron-sufficient mice, was much lower after dosing with nano Fe(III) than ferrous sulfate (Figure 1). Finally, we have validated in the anemic CD1 mouse, which is commonly used in iron metabolism studies, that nano Fe(III) is very similar in efficacy to the clinical standard ferrous sulfate for restoring hemoglobin concentrations (Figure 1).

Tissue nonheme-iron concentrations in the liver and spleen of mice were reduced after 3 wk of dietary-induced iron deficiency (Figure 2) showing that these mice had indeed low iron status (37). Mean hepatic, splenic, and duodenal nonheme-iron concentrations were similar after supplementation with nano Fe(III) or FeSO_4_ (Figure 2). After the 7-d supplementation period with nano Fe(III) or FeSO_4_, hemoglobin concentrations and iron stores in the mice were still suboptimal, and this was most substantial in the spleen (Figure 2).

We previously reported, using chemical inhibitors, that nano Fe(III) is taken up apically in Caco-2 cells by endocytosis followed by pH- and ligand-driven breakdown in endosomes or lysosomes within the cell (19). Although our data alluded to endocytosis as the uptake mechanism, it remains unresolved as to whether the process is by pinocytosis or by a receptor-mediated route that may resemble that proposed for dietary ferritin uptake (38). The current study adds further evidence for a distinct mechanism of cellular uptake of nano Fe(III) compared to that of soluble iron. We used molecular techniques, notably inhibition of DCYTB activity and *Slc11a2* knockdown with siRNA, in preference to the less-specific pharmacologic inhibitors and have again shown that nano Fe(III) does not require redox activity at the mucosal surface of the cell before uptake and that the uptake is independent of DMT1 (Figure 3). We believe that an independent mechanism is now proven for the uptake of nano Fe(III) by enterocytes because both approaches, namely pharmacologic and molecular, lead to the same conclusions in vitro [Figure 3 (19)] and are supported by chemical inhibition studies in vivo (18). Of course, although the apical uptake of nano Fe(III) might be independent of DMT1, the intracellular trafficking of Fe derived from this compound, before basolateral export from the enterocyte, may still involve vesicles containing internalized DMT1 just as is hypothesized for iron from soluble sources (39, 40). This could be best investigated with use of the intestinal-specific DMT1-knockout mouse model (41) and intracellular imaging of iron because the formation of intracellular ferritin in cell culture, as studied here, does not seem to require DMT-1.

Taken together, our data demonstrate that nano particulate tartrate-modified Fe(III) poly oxo-hydroxide [i.e., nano Fe(III)] is equally bioavailable to FeSO_4_ but that its apical cell uptake is independent of DCYTB/DMT1-mediated import of soluble iron. In our view, this distinct mechanism offers 2 main advantages: *1*) no requirement for reduction of Fe(III) at the mucosal surface and, therefore, nano Fe(III) should have restricted luminal and mucosal redox activity (and thus toxicity) (42, 43) while *2*) nano Fe(III) absorption is unlikely to be influenced by other divalent metals present in the diet such as Zn or, indeed, vice versa (44–46). Furthermore, any unabsorbed nano Fe(III) should have limited redox activity in the distal colon because of the spatial arrangement of the iron in the nanoparticle, which renders the generation of oxygen radicals less probable (47). Utilization by the gut microbiota of iron from nano Fe(III) would also be more challenging (48, 49).

Therefore, nano Fe(III) presents as a unique, safe, and bioavailable novel form of oral iron for use in the treatment of IDA.

## Footnotes

5 Abbreviations used: CD1, outbread Swiss mouse strain; DCYTB, duodenal cytochrome b; DMT1, divalent metal transporter 1; FAC, ferric ammonium citrate; Fe-NTA, ferric nitrilotriacetate chelate; FeSO_4_, ferrous sulfate; *Hamp1*, hepcidin antimicrobial peptide 1; IDA, iron deficiency anemia; *Rpl19, *mouse ribosomal protein L19; siRNA, small interfering RNA; *Slc11a2*, solute carrier family 11 (proton-coupled divalent metal ion transporter), member 2.
